# Survey of Water Bugs in Bankim, a New Buruli Ulcer Endemic Area in Cameroon

**DOI:** 10.1155/2012/123843

**Published:** 2012-05-16

**Authors:** Solange Meyin A. Ebong, Sara Eyangoh, Estelle Marion, Jordi Landier, Laurent Marsollier, Jean-François Guégan, Philippe Legall

**Affiliations:** ^1^Service de Mycobactériologie, Centre Pasteur du Cameroun, Cameroun-Réseau International des Institut Pasteur, BP 1274 Yaoundé, Cameroon; ^2^Biodiversité et Évolution des Complexes Plantes-Insectes Ravageurs-Antagonistes UR-072, Institut de Recherche pour le Développement Cameroun, Yaoundé, Cameroon; ^3^UMR MIVEGEC IRD, CNRS, Universités de Montpellier 1 et 2, Centre IRD de Montpellier, Montpellier, France; ^4^Centre de Recherche sur le Cancer Nantes-Angers, LUNAM, Université de Nantes and Université d'Angers, 49000 Angers, France; ^5^UMR 892, Inserm, 49000 Angers, France; ^6^UMR 6299, CNRS, 49000 Angers, France; ^7^Groupe d'Etude des Interactions Hôte-Pathogène, Université d'Angers, 49000 Angers, France; ^8^Unité d'Epidémiologie des Maladies Emergentes, Institut Pasteur, Paris, France; ^9^Ecole des Hautes Etudes en Santé Publique, Centre Interdisciciplinaire BIODIV, EHESP, Montpellier, France; ^10^Institut de Recherche pour le Développement (IRD), UR 072, BP1857, Yaoundé, Cameroon; ^11^Laboratoire Evolution, Génomes et Spéciation, UPR 9034, Centre National de la Recherche Scientifique (CNRS), 91198 Gif sur Yvette Cedex, France; ^12^Université Paris-Sud 11, 91405 Orsay Cedex, France

## Abstract

Buruli ulcer is a debitliating human skin disease with an unknown transmission mode although epidemiological data link it with swampy areas. Data available suggest that aquatic insects play a role in the dissemination and/or transmission of this disease. However, their biodiversity and biology remain poorly documented. We conducted an entomological survey in Bankim, Cameroon, an area recently described as endemic for Buruli ulcer in order to identify the commonly occurring aquatic bugs and document their relative abundance, diversity, and spatial distribution. Collection of aquatic bugs was realized over a period of one month by daily direct capture in different aquatic environments (streams, ponds, and rivers) and through light traps at night. Globally, the data obtained showed the presence of five families (Belostomatidae, Naucoridae, Nepidae, Notonectidae, and Gerridae), their abundance, distribution and diversity varying according to the type of aquatic environments and light attraction.

## 1. Introduction

Buruli ulcer is a debilitating human skin disease caused by *Mycobacterium ulcerans *[1, 2]. This infection is a neglected emerging disease that has recently been reported in some countries as the second most frequent mycobacterial disease in humans after tuberculosis [3, 4]. The majority of cases are localized in Africa occurring mainly in poor local communities. Other cases have been reported in Asia, Australia, and South America [1].

Despite the increasing number of endemic areas, the exact mode of transmission of *M. ulcerans* remains unclear. Buruli ulcer has always been associated with swampy areas [5–10]. The role of aquatic insects in Buruli ulcer transmission has been suggested by some studies in the past 10 years [11, 12]. Experimental laboratory studies have confirmed this possibility by showing that *M. ulcerans* was able to settle in glands of water bugs and transmitted to mice through bitings. Field investigations found that water bugs captured in endemic areas were positive for *M. ulcerans *[13–19]. Recently, viable *M. ulcerans* was detected in saliva of water bugs [20]. These studies allowed proving that water bugs are the host and a probable vector of* M. ulcerans*. However, the exact role of aquatic bugs remains to be clarified. Indeed, the etiological agent of Buruli ulcer may be introduced by bites of these insects or by trauma at skin sites [11, 12, 21].

All over the world, about 45000 species of insects are known to inhabit diverse freshwater ecosystems [22, 23]. These insects are involved in nutrient recycling and form an important component of natural food webs in aquatic ecosystems [24]. They also serve as reliable indicators of ecological characteristics of water [25]. Among aquatic insects, water bugs belong basically to two categories: semiaquatic bugs which live upon the water surface and true water bugs which live beneath the water surface. Most of them are carnivorous and can even feed on small vertebrates such as fishes and amphibians [26–28]. The majority of water bug species are also known to display flying activity, in their adult forms at night when attracted to light [29–32]. They may therefore also play a role in *Mycobacterium ulcerans *dissemination in the environment as suggested by [20, 33–35].

Bankim district has been described recently as a Buruli ulcer endemic site in Cameroon, and aquatic bugs collected in this region were positive for *M. ulcerans *[34]. But aquatic bugs' biodiversity and biology are poorly documented, making it hard to characterize the relations between *M. ulcerans* and these aquatic insects. In the above-mentioned context, the present study was carried out with 2 objectives:

1. to identify the commonly occurring medium and large size aquatic bugs fauna elements; to work out their relative abundance, diversity; to perform comparison between daytime square-net captures and night time light trap captures;
2. to provide a database and spatial distribution of aquatic bugs related with Buruli ulcer cases in this area.

## 2. Materials and Methods

### 2.1. Sites of Study

**Figure 1:**
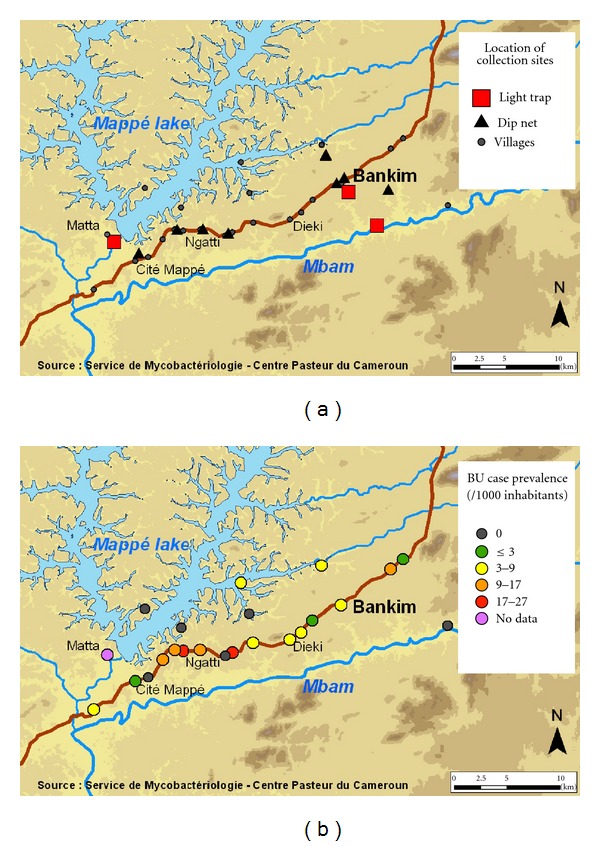
(a) Study site; (b) Buruli ulcer case prevalence per village.

Capture of aquatic bugs was organized within June 2009 in Bankim (6.0405N 10.2737E), a rugged land in north-western Cameroon at an altitude of about 750 meters. This region represents a transition between forested south and savanna north. Its geography, tropical climate and population contexts differ from the forested Nyong River Basin, the endemic region of Central Cameroon. The building of a dam on the Mape River in 1989 profoundly modified the environment by creating an artificial lake of 3.2 billion m³ capacity. This study was carried out in selected water bodies (5 streams, 3 ponds, 1 river) and in different geographic landscapes like the savanna around the dam, near the habitations, and the forest around the Mbam River. Water bodies were located around Bankim town, in Ngom and along the Bankim Mappé road (Figure 1(a)). The incidence of Buruli ulcer in this region is increasing [34]. Farming is the main activity with specific population groups raising cattle and other involved in commercial fishing. The population density is about 30 inhabitants/km². The prevalence of Buruli ulcers in this endemic area is represented in Figure 1(b).

### 2.2. Aquatic Bug Capture and Sampling

The medium and large size water bugs were collected using two sampling methods: direct method in aquatic environment and indirect method by using light trap to capture winged imagos.

Direct collection of insects was performed daily for a period of one month in water bodies. Sampling was made by hauling a square-net (32 × 32 cm and 1 mm in mesh size) from the surface to a *depth* of 1 meter and over a distance of 1 meter. A given sample corresponds to the mixture of all insects collected after 45 minutes. After collection, insects were transported in labelled plastic bottles containing freshwater from the site. Adults as well as nymphs were then selected, counted, and preserved in 70% ethanol for laboratory identification. For each site, GPS coordinates, nature, and intensity of human activities in water, type of water body were noted.

Night time light trapping was used for indirect insect collection. This mobile light trap consisted of a 250 W bulb connected to an electrical generator put in front of a white sheet. Light traps were installed, respectively, five times around the dam and the Mbam River and 4 times near habitations from 6:30 PM to 11:00 PM beginning at full moon and ending at the end of lunar cycle. All attracted insects were collected in labelled plastic bottles containing 70° ethanol and processed as indicated previously [20].

Three sites were selected for night time collection, one by the forest zone Matta, another in the savannah Bankim, and the last one near habitation.

### 2.3. Water Bugs Identification

Aquatic Heteroptera, generally called water bugs, forms three infraorders of Hemiptera order (Leptopodomorpha, Geromorpha, and Nepomorpha) which belong to the Insect class and the Arthropoda phylum. Heteroptera is mainly identified by observing:

1. piercing-sucking mouthparts, with a segmented rostrum arising from the front of the head;
2. two pairs of wings in adults: partly membranous forewings; hemelytra and fully membranous hind wings.

Identification of water bugs took place in the entomological laboratory of the Institute of Agricultural Research for Development (IRAD) in Yaoundé Cameroun. Each collected specimen was attributed to a given family on the basis of the Heteroptera family determination criteria [36]. Currently, identification keys enable identification only for water bug families.

## 3. Results

### 3.1. Direct Insect Collection

**Figure 2:**
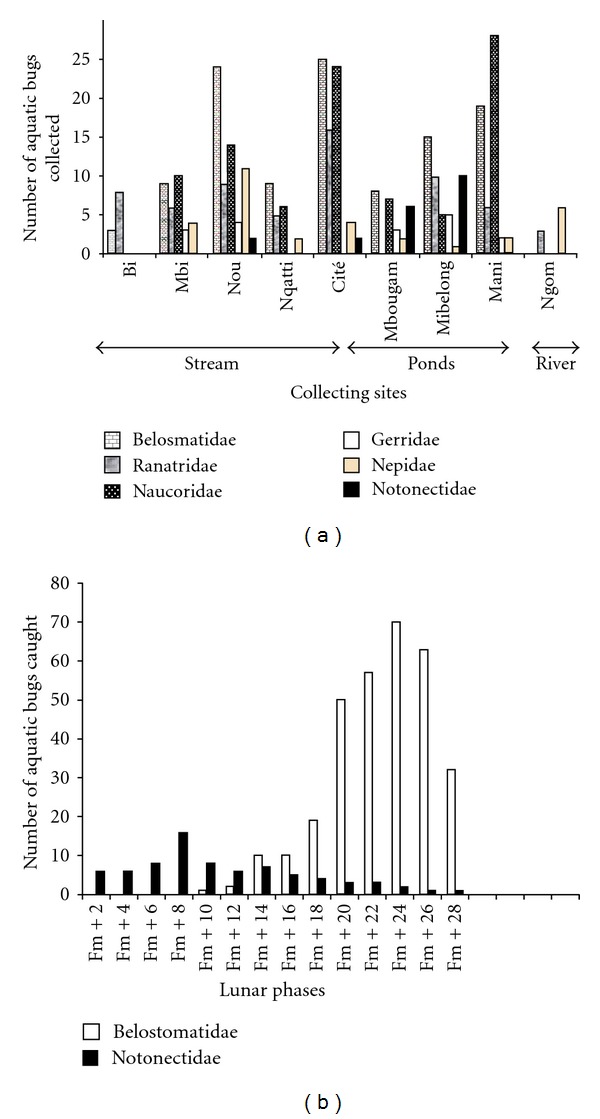
(a) Aquatic bugs per family and collecting water bodies in the aquatic environment. (b) Aquatic bugs caught in a light traps per families according to lunar phases.

Globally, 728 water bugs were collected, within which 338 were collected directly in aquatic environments and 390 captured through light traps. Those collected in aquatic environment belong to five families (Belostomatidae 33.13% (*N* = 112), Naucoridae 27.81% (*N* = 94), Nepidae 28.09% (*N* = 95), Notonectidae 5.91% (*N* = 20), and Gerridae 5.02% (*N* = 17)). But their abundance, distribution, and diversity vary according to the type of water body. The river was poor in aquatic bug population, with only one family captured; Nepidae (*N* = 9) represented by two subfamilies Ranatrinae (*N* = 3) and Nepinae (*N* = 6). Generally, in the ponds and streams, the five families were present but their abundance and diversity seem only dependent on the geographical location of the collecting site as on Figure 2(a). 

### 3.2. Indirect Insect Collection

**Figure 3:**
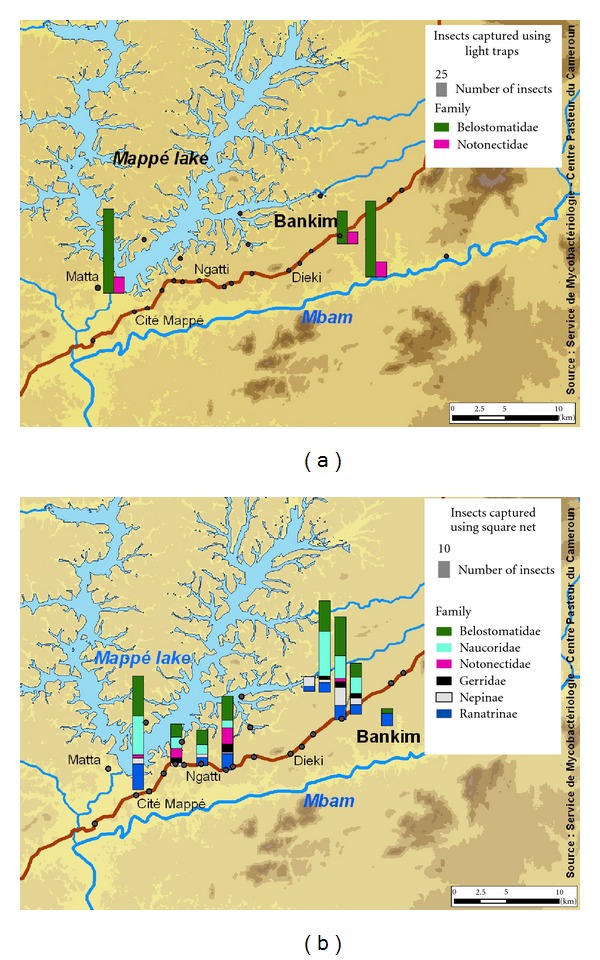
(a) Distribution of Hemiptera captured in the night by light traps; (b) distribution of Hemiptera collected directly in the water bodies by square-net.

Light trap indirect collection yielded 390 specimens belonging only to 2 families; Belostomatidae represented 80.51% and Notonectidae 19.48% (Figure 2(b)). During the full moon, only the Notonectidae family come to light; Belostomatidae were absent at this phase of the, but they appeared a few nights after (Figure 2(b)). Belostomatidae family was very abundant mainly during the few nights that precede or follow the full moon, showing several peaks, which decreased progressively for rescinding at a few nights before full moon. Whatever the site of collection, the numerical variations of water bugs captured by light trap were almost consistent with Belostomatidae being prominent, that is, 33.33% of Belostomatidae and 5.12% of Notonectidae in the forest; 25.64% of Belostomatidae and 11.94% of Notonectidae in the savanna; 21.53% of Belostomatidae and 2.56% of Notonectidae near habitations (Figure 3(a)). 

## 4. Discussion

### 4.1. Abundance Variation according to Type of Water Bodies

We noted that streams and ponds which were slow and stagnant showed the highest number of water bugs: 59.17% in the streams and 38.16% in the ponds. The number of individuals was quasinil in the river; only 9 (2.66%) water bugs were collected in the river perhaps because of its rapid flow. Furthermore, aquatic vegetation was abundant in streams and ponds, but it was scarce in the river. In all water bodies selected, we observed human activities but we cannot say if they influenced or not diversity of aquatic bugs. Nevertheless, it seems that diversity of water bugs was related with nature of water currents and presence of aquatic vegetation.

Spatial distribution of aquatic bugs is not uniform and does not depend on the type of water body but on the geographical location as illustrated in Figures 2(a) and 3(b). For example, Notonectidae family was met in only 2 of the five streams and 2 of the 3 ponds. Belostomatidae and Naucoridae were found in all ponds and streams. These last two families (Belostomatidae and Naucoridae) are carnivorous and suspected to play a role in the transmission of Buruli ulcer and in the ecological expansion of the *Mycobacterium ulcerans *niche.

### 4.2. Abundance Variation according to Moon Cycle

During the moon cycle, Notonectidae family was present at all times but in less important numbers. These results agreed with the results obtained concerning flight activity of Belostomatidae [26]. Light trapping proved to be an interesting method to obtain important numbers of Belostomatidae and Notonectidae but reflected poorly the overall diversity of water bugs.

The number of water bugs is more important around the Mbam River which was situated in the forest than the other sites installed light trap. The installation near the habitations showed the least number of aquatic bugs. These results concerning the abundance of water bugs in the area neighbouring the Mbam River are to be related with the results of an epidemiological survey performed simultaneously [10]. In this case-control study, having baths for hygiene in the Mbam River was shown to increase the risk of Buruli ulcer in the populations odds ratio (95% confidence interval) = 6.9 (1.4 − 35).

### 4.3. Seasonal Variations

**Figure 4:**
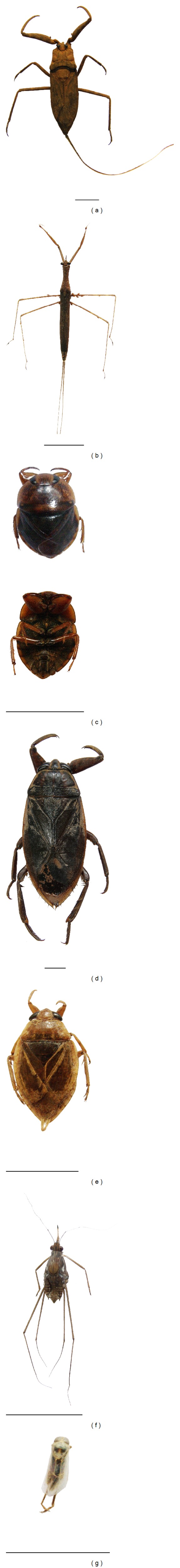
Specimens of water bugs collected during the study. (a) Nepinae, (b) Ranatrinae, the two subfamilies of Nepidae, (c) Naucoridae family (dorsal and ventral views), two morphotypes Belostomatidae, (d) giant Belostomatidae, (e) small size Belostomatidae, (f) Gerridae, and (g) Notonectidae.

Water bugs collection conducted in Bankim during the rainy season in June permitted us to identify five water bug families: four families of true water bugs and one semiaquatic bugs (Table 1). These families include many unknown species as determination keys for water bugs species are not yet available for West Africa. These results are low comparing with those in another study in the same region during the long dry season in January which showed 1349 specimens belonging eight families [34]. In this study, *Mycobacterium ulcerans* molecular signatures were searched; among 244 insect pools (pool = group of ten insects belong the same family), 12 (5%) were M*. Ulcerans* positive. *M. ulcerans*-positive saliva was found in 11 (18%) of 61 insects in the family Belostomatidae and in 3 (8%) of 38 in the family Naucoridae. Beyond number of families in two studies, a large difference in numerical data of insect specimens was observed, 728 during the rainy season against 1349 in the long dry season. More sampling is required to confirm these results. All water bugs families collected in this study (Figure 4) were found in Akonolinga, the other Buruli ulcer endemic area in Cameroon [20].

## 5. Conclusion

This preliminary entomological survey in Bankim shows the distribution and diversity of aquatic bugs colonization of water bodies and reveals that their flight activity is influenced by light (direct and moon light). It also shows that the diversity of water bugs depends partly on the types of water bodies in the same endemic area, with streams and ponds seeming to be selective habitats offering best life conditions. Light attraction and the moon phases appeared to be influencing factor for aquatic bug's distribution. In prospective, this preliminary results need to be confirmed through monthly collection in endemic and nonendemic areas. Moreover, detection of *M. ulcerans *in salivary glands of the water bugs, in particular those are able to bite humans (Belostomatidae and Naucoridae), will support their involvement in ecology and transmission of *M. ulcerans* [20].

## Figures and Tables

**Table 1:** Identification of water bugs collected directly in aquatic environment and indirectly by light trap.

Category	Family	Sub family	Genus	Direct collection	Indirect collection
True water bugs	Belostomatidae	Belostomatinae	Appasus	112	114
	Belostomatidae	Lethocerinae	Lethocerus	0	200
	Nepidae	Rantrinae	ND	63	0
	Nepidae	Nepinae	ND	32	0
	Naucoridae		ND	94	0
	Notonectidae	Anisopinae	ND	14	46
	Notonectidae	Notonectinae	ND	6	30

Semiaquatic bugs	Gerridae	Gerrinae	ND	17	0
